# Electrospinning Membrane with Polyacrylate Mixed Beta-Cyclodextrin: An Efficient Adsorbent for Cationic Dyes

**DOI:** 10.3390/polym17020243

**Published:** 2025-01-20

**Authors:** Chunling Zheng, Wei Zhao, Xiaoqian Tu, Shaoqiang Zhou

**Affiliations:** 1 College of Food Science and Light Industry, Nanjing Tech University, 30 South Puzhu Road, Nanjing 211816, China; 2 Institute of Textile Auxiliary and Ecological Dyeing Finishing, Nanjing Tech University, 30 South Puzhu Road, Nanjing 211816, China; 3 Jiangsu Textile Product Quality Supervision and Inspection Institute, 3 East Guanghua Street, Nanjing 210007, China; 4 Nanjing Customs Industrial Products Testing Center, 39 Chuangzhi Road, Nanjing 210019, China

**Keywords:** *β*-cyclodextrin, polyacrylate, electrospinning, electrospinning, adsorption, cationic dye

## Abstract

A simple and non-chemical binding nanofiber (*β*-CD/PA) adsorbent was obtained by electrospinning a mixture of *β*-cyclodextrin (*β*-CD) and polyacrylate (PA). The cationic dyes in wastewater were removed by the host–guest inclusion complex of the *β*-cyclodextrin and the electrostatic interaction between the polyacrylate and the dyes groups. The influence of the content of *β*-cyclodextrin on the surface morphology and adsorption capacity of the nanofiber membrane was discussed, and the optimized adsorption capacity of nanofiber adsorption material was determined. The adsorption capacity of nanofiber adsorbents for basic red 9, basic red 14, basic red 46, basic blue 9, basic yellow 19 and basic yellow 28 was 86.71 mg/g, 21.513 mg/g, 18.926 mg/g, 44.525 mg/g, 116.516 mg/g and 155.206 mg/g, respectively. The effects of different initial concentrations and pH values on the adsorption properties of adsorbent materials were studied. The kinetic analysis showed that the adsorption process of nanofibers for cationic dyes was more in line with the pseudo-second-order kinetic adsorption model. Moreover, nanofiber adsorbent could be easily separated from the dye solution and showed high recycling efficiency. These results indicated that the *β*-cyclodextrin/polyacrylate composite nanofibers are expected to be recyclable adsorbents in dye wastewater treatment.

## 1. Introduction

Water pollution has always been a serious environmental problem. The printing industry and the textile industry produce a large amount of dye wastewater, which is one of the main sources of water pollution [1,2,3]. If not properly treated, direct discharge into the environment will bring many environmental problems. Various methods, including electrochemical, photocatalytic degradation, adsorption and ultra-filtration, have been widely investigated for treating dye polluting wastewater [4,5,6,7,8]. Some studies have shown that the adsorption method has advantages of high efficiency, economy, simplicity, and good selectivity in treating dye wastewater [9,10,11]. There are many ways to prepare adsorbent materials, and electrostatic spinning is one of the common methods for preparing high-efficiency adsorbent materials [12]. Multifunctional nanofibers can be electro-spun by using polymer composites or polymer blends that have many unique advantages, such as relatively high surface area and porosity, and fiber diameters at the nanometer scale [13,14]. Therefore, the electrospinning technology is used in many fields, including biological materials, filtration membranes, electroanalysis, etc. [15,16,17]. In addition, the adsorption effect of electro-spun nanofiber membranes is superior to conventional adsorption membranes because they can be easily designed to obtain functional surface adsorption sites or specific structures, which are beneficial for providing better adsorption capacity [18].

*β*-cyclodextrin (*β*-CD) is a cyclic oligosaccharide formed by the linkage of seven glucose monomers via *α*-1,4 glycosidic bonds. *β*-CD has a special molecular configuration: the whole molecule looks like a conical ring with some hydrophilic hydroxyl groups on the outside and a hydrophobic cavity inside. Due to its special molecular configuration, *β*-CD can form a non-covalent inclusion compound with some compounds [19,20]. Therefore, *β*-CD can also be used as an adsorbent for treating dye wastewater, because it can form inclusion complexes with dye molecules in contaminated wastewater [21,22].

For instance, Fan et al. [5] obtained a grafted product of cyclodextrin and chitosan (*β*-CD–chitosan) by connecting one end of maleic anhydride to the hydroxyl group at the sixth position of cyclodextrin and the other end to chitosan. Then, *β*-CD–chitosan and magnetic Fe_3_O_4_ were combined under the action of glutaraldehyde to form magnetic modified chitosan nanoparticles. It was found that this nanoparticle material had a good adsorption capacity for methyl blue. Ebadi et al. [19] self-assembled mesoporous silica particles using cetyltrimethylammonium bromide, ammonia water and ethyl silicate. Then, these particles were combined with 3-aminopropyltriethoxysilane in toluene to form amino-functionalized silica particles. Subsequently, the amino-functionalized particles were conjugated with *β*-CD modified by p-toluene-sulfonyl chloride to obtain a functionalized silica particle (*β*-CD-SNHS) adsorbent with multiple cyclodextrins, which was significantly superior to other existing adsorbents in removing methylene blue from aqueous solutions.

However, as a powder solid, *β*-CD is difficult to be used in water treatment. In recent years, some researchers have prepared a nanofiber membrane with water resistance by electrospinning a mixture of *β*-CD and some high molecular polymers (dissolving *β*-cyclodextrin and high molecular polymer in an organic solvent) [14,23], in order to achieve synergistic adsorption of the dye molecules by the *β*-CD and the polymer [2]. The copolymer of methyl methacrylate and butyl acrylate has characteristics of good water resistance, good toughness, strong compatibility and easy preparation, and the properties of the copolymer can be changed by adjusting the ratio of the monomer [24,25].

In this research, polyacrylate (PA) was synthesized by emulsion polymerization with methyl methacrylate, butyl acrylate, and acrylic acid as monomers, and the adsorption materials of cationic dyes were prepared by electrospinning a mixture (DMF as solvent) of cyclodextrin and polyacrylate. The effect of *β*-CD content (mass ratio of *β*-CD to PA) on the morphology and adsorption properties of electro-spun fibers was investigated, and the nanofiber materials with the best adsorption effect were determined. The distribution of cyclodextrin in fiber materials was analyzed by XRD. The adsorption properties of fiber materials at different initial solubility and pH were discussed experimentally, and the kinetics of the adsorption process was also studied. Finally, the desorption capacity and recycling efficiency of the fiber material were tested. The dynamic process of the possible adsorption and desorption of the nanofiber materials was shown.

## 2. Experimental

### 2.1. Materials

**Figure 1:**
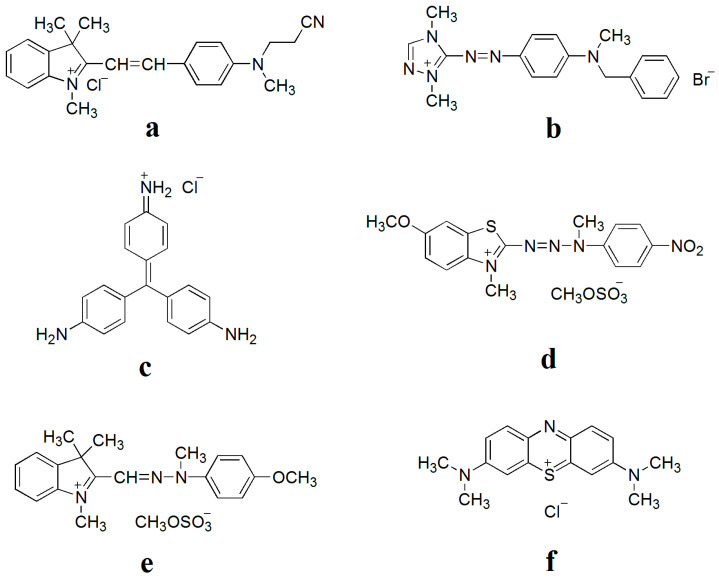
Molecular structures of basic red 14 (**a**), basic red 46 (**b**), basic red 9 (**c**), basic yellow 19 (**d**), basic yellow 28 (**e**) and basic blue 9 (**f**).

*β*-cyclodextrin (*β*-CD), potassium peroxydisulfate (K_2_S_2_O_8_), OP-10, sodium dodecyl sulfate (SDS), methyl methacrylate (MMA), butyl acrylate (BA), acrylic acid (AA) and N,N-dimethylformamide (DMF)were purchased from LingFeng Chemical Inc., Shanghai, China. Cationic dyes were obtained from XingWu Chemical Industry Co., Ltd., Rugao, Jiangsu, China. The structures of six cationic dyes, including basic red 14, basic red 46, basic red 9, basic yellow 19, basic yellow 28 and basic blue 9, are shown in Figure 1 [26]. All chemicals are analytical grade and used without further purification and all the solutions are prepared with homemade deionized water.

### 2.2. Synthesis of Polyacrylate (PA) Copolymer

90 mL deionized water, 0.9 g OP-10, and 0.45 g SDS were added into the beaker and stirred fully, and 21 g MMA, 19 g BA, and 5 g AA were added continuously and stirred well. The mixture in the beaker was placed under a high-speed rotating homogenizer at 14,000 r/min, and a milky emulsion was obtained after high-speed homogenization for 6 min.

A proportion of 2/3 emulsion was poured into a constant pressure drip hopper, and the remainder was heated in a four-mouth flask to 75 °C. K_2_S_2_O_8_ was added to the reaction emulsion as an initiator and the timing began at this point as the reaction starting point. A period of uniform stirring made the reaction emulsion pale blue, then the reserved 2/3 emulsion kept dribbling to the flask slowly at a constant speed, for around 2 h. After the pre-emulsion was added, K_2_S_2_O_8_ continued to initiate the subsequent polymerization at a strictly controlled 75–80 °C for 1 h until the system temperature reached 85 °C, and insulation needed another 1 h. Finally, the target emulsion was cooled to room temperature and filtered with a Buchner funnel.

### 2.3. Preparation of Electrospun β-CD/PA Nanofiber Membrane

The polyacrylate emulsion was taken into the evaporating dish and placed in the oven to obtain a colorless and transparent copolymer solid. An electro-spun solution consisting of 2 g of the copolymer solid and a different experimental quantity of *β*-CD dissolved in 18 mL DMF was mixed mechanically with stirring for 10 h. A 10 mL plastic syringe with a metal needle was filled with the resulting mixture and carried forward at an optimized speed of 1.5 mL/h during the electrospinning. A charged voltage of 13 kV was loaded between the needle and the grounded metal collector covered with a sheet of aluminum foil, which was served as a counter electrode. The distance between the needle and the collector was 12 cm. The electrospinning process was performed at relatively low humidity (40–60%) at room temperature.

### 2.4. Characterization

The scanning electron microscope (S-3400N II, Hitachi Production Co., Ltd., Shanghai, China) was used to observe the morphology of the *β*-CD/PA fiber membrane, and the samples were gold sputter coated to guarantee the stability of SEM imaging. XRD diffraction of the samples was obtained by an X-ray diffraction spectrometer (D8 Advance, Bruker Corporation, Beijing, China) using Cu-K*α* radiation. The physical properties of the electrospinning solution were measured by conductivity meter (DDS-307A, INESA Instruments Co., Ltd., Shanghai, China) and viscosity meter respectively (SNB-3, Shanghai Precision Instrument Co., Ltd., Shanghai, China). The adsorption number of nanofibers could be obtained via a difference method by measuring the concentrations of dyes solution before and after adsorption with ultraviolet spectrophotometer (Cary 60, Agilent Technologies (China) Co., Ltd., Beijing, China).

### 2.5. Batch Adsorption Experiments

All experiments were carried out at room temperature and all adsorption processes were static adsorption. The absorbability of nanofibers was studied first. A total of 30 mg *β*-CD/PA nanofiber membrane with different *β*-CD contents was suspended in 20 mL dye solution (100 mg/L) of basic red 14 or basic yellow 28 to compare their respective adsorption effects. The maximum adsorption capacity of nanofibers to various cationic dyes was obtained when adsorption reached equilibrium. Next, the pH of the dye’s solution was adjusted with 1.0 M sulfuric acid or sodium hydroxide to witness the effect of pH on the adsorption of nanofiber membrane. Finally, the adsorption kinetics were researched. The typical batch adsorption experiment was carried out as follows: 50 mg nanofiber membrane was placed in 20 mL basic yellow 28 (50 mg/L), basic yellow 19 (30 mg/L), basic red 46 (15 mg/L), basic red 14 (15 mg/L), basic blue 9 (10 mg/L) and basic red 9 (10 mg/L) solutions, respectively, and the adsorption capacity was measured periodically.

The adsorption capacity of the nanofiber membrane is calculated according to the concentration difference of dye solution before and after adsorption, and the calculation formula is:

(1) $$q_{e}=\left(C_{i}-C_{e}\right)V/M_{S}$$

where *q*_e_ (mg/g) is the adsorption capacity, *C*_i_ (mg/L) is the initial concentration of dye solution, *C*_e_ (mg/L) is the equilibrium concentration of dye solution after adsorption, *V* (L) is the volume of dye solution and *M*_s_ (g) is the mass of solid adsorbent.

## 3. Results and Discussion

### 3.1. Determination of Optimum Adsorption Capacity of Nanofibers

**Figure 2:**
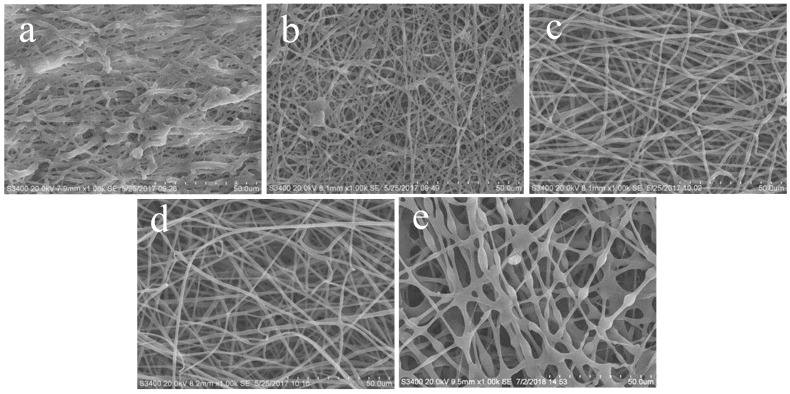
SEM images of PA electro-spun nanofibers without *β*-CD (**a**); *β*-CD/PA composite electro-spun nanofibers with *β*-CD mass accounting for 25% (**b**), 50% (**c**), 75% (**d**) and 100% (**e**) of PA quality.

During the electrospinning process, the concentration of PA was kept at 12% (Mass of PA/DMF volume, g/mL), and the mass of *β*-CD added to the solution was changed to obtain five different spinning fluids. The conductivity and viscosity of the five spinning fluids and the diameter of five nanofibers are shown in Table 1. Five different nanofiber membranes were obtained by these spinning fluids through electrostatic spinning, as shown in Figure 2.

The morphology of the electro-spun fiber can be observed in Figure 2. When the content of *β*-CD increased from 0 to 25% (Figure 2a,b), the filamentous shape of the spun fiber went from uneven to uniform and the diameter of the fiber decreased considerably. This indicated that the introduction of *β*-CD improved the viscosity of the system and significantly enhanced the filament formation of the nanofibers. When the content of *β*-CD increased to 50% (Figure 2c), the beads on the surface of the nanofibers decreased significantly, the individual fibers became more uniform and the gap between the fibers was more obvious. As the content of *β*-CD approached 75% (Figure 2d), the morphology of the nanofiber did not change significantly, but the diameter of some nanofibers continued to decrease and the gap between the fibers kept widening. Therefore, keeping the content of *β*-CD in the spinning liquid system between 50% and 75%, the silky uniform nanofibers could be favorably obtained. When the *β*-CD content reached 100% (Figure 2e), the viscosity of the spinning solution increased but the conductivity decreased, which reduced the charge density during the electrostatic spinning and caused the electrostatic repulsion on the surface of droplet. As a result, the draft ratio of jet flow decreased to make the diameter of the electro-spun fiber thicker and beads appear.

**Figure 3:**
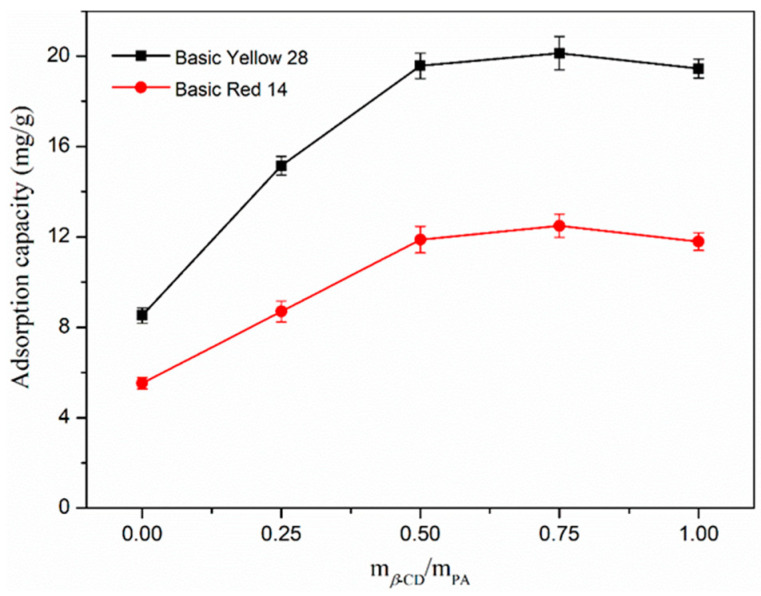
Adsorption of *β*-CD/PA nanofiber membrane with different *β*-CD content for basic yellow 28 (black line) and basic red 14 (red line).

The adsorption for two cationic dyes were tested by using nanofiber membranes with different *β*-CD contents of the same quality under the same conditions, and the results are shown in Figure 3. With an increasing amount of *β*-CD, the adsorption of the nanofiber membranes performed increasingly well, and reached a peak when the content of *β*-CD was 75%. This showed that the participation of *β*-CD not only improved the morphology of the nanofiber but also increased its adsorptive property for cationic dyes. However, the excessive *β*-CD did not further perpetuate this effect, which might make spinning difficult and the diameter of nanofibers large and uneven. As a result, the contact sites among the dye molecules and the nanofibers reduced. Taken together, when the content of cyclodextrin reached 75%, nanofibers with uniform morphology and maximum adsorption capacity could be obtained.

**Figure 4:**
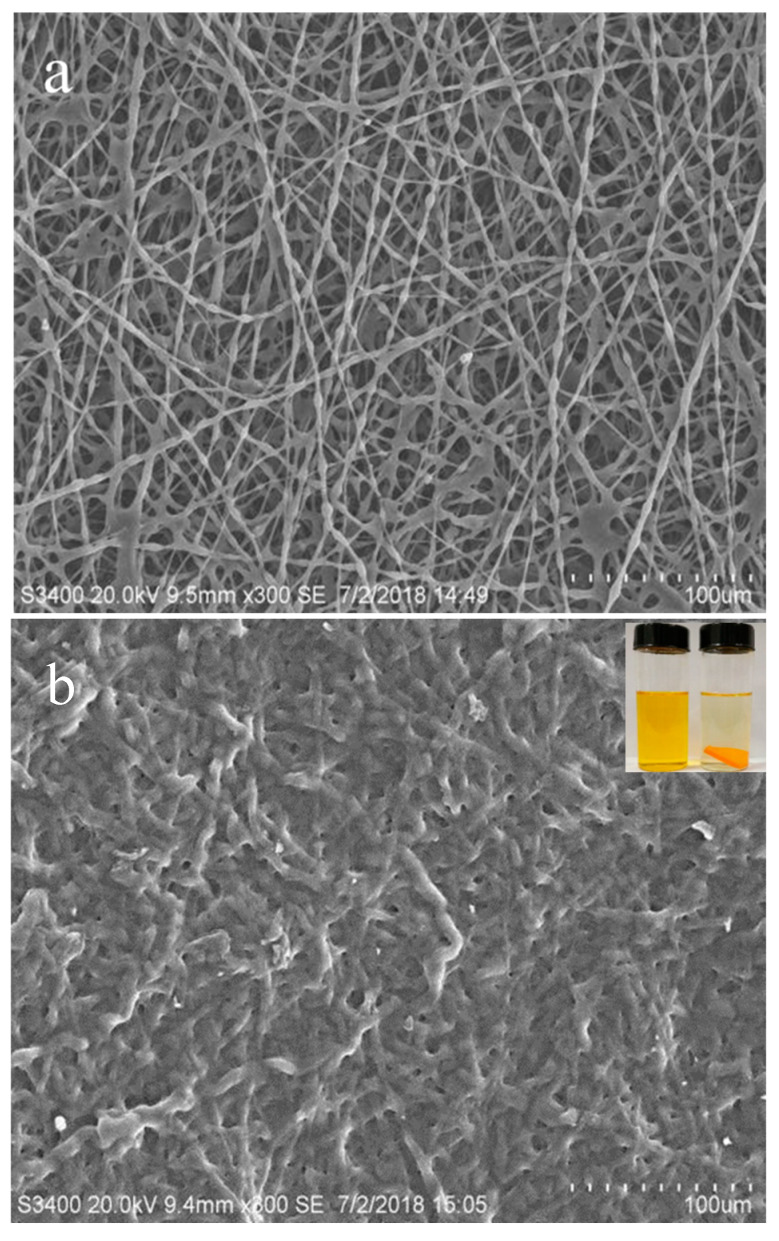
SEM images of nanofibers before (**a**) and after (**b**) adsorbing basic yellow 28.

Figure 4 shows the SEM images of nanofiber membrane before and after adsorption for basic yellow 28. It is obvious in Figure 4b that the adsorbed nanofibers have a large amount of cationic dye enrichment, indicating that the prepared adsorbent had good adsorption for cationic dyes. At the same time, because of the spatial size and steric resistance of the long side chain of the basic red 14 molecule, the difficulty of *β*-CD inclusion is obviously greater than that of the basic yellow 28 molecule. Therefore, during the adsorption process, the adsorption capacity of basic yellow 28 is significantly higher than that of basic red 14.

### 3.2. XRD Characterization of the Obtained Nanofibers

**Figure 5:**
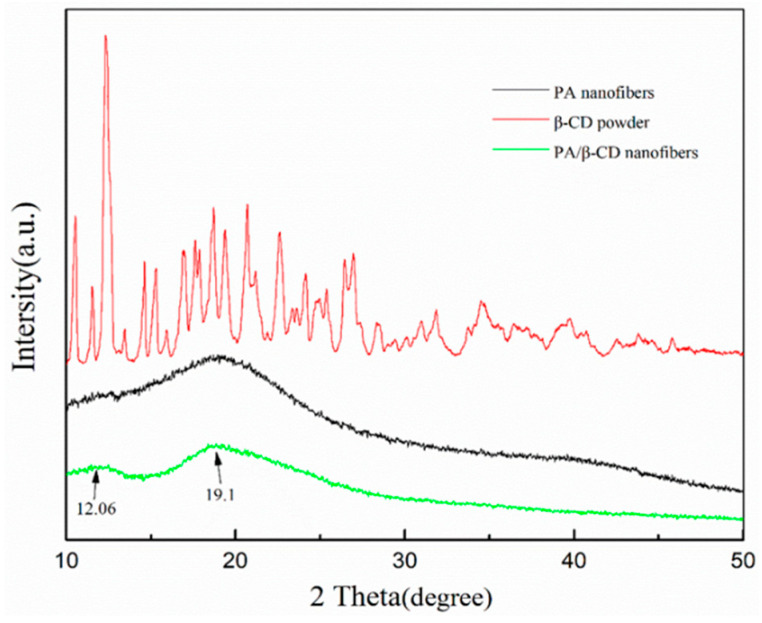
XRD of *β*-CD powder, PA nanofiber, and PA/*β*-CD nanofiber.

In order to better explore the crystallization and distribution of cyclodextrin in nanofiber, the XRD diffraction analysis was performed on *β*-CD powder, PA nanofiber, and PA/*β*-CD composite nanofiber, as shown in Figure 5.

It can be seen from the figure that, in the range of 2θ from 10° to 50°, the *β*-CD powder had many strong diffraction peaks, but the PA nanofiber had a broad diffraction peak only at 19.1°. The XRD of the PA/*β*-CD nanofiber membrane is similar to that of the polyacrylate film, which indicated that the *β*-CD molecules should be uniformly distributed in the composite nanofibers in an amorphous form. The difference between the XRD of the PA/*β*-CD nanofiber and the PA nanofiber was that the PA/*β*-CD nanofiber membrane exhibited a new diffraction peak at 2θ of 12.06° with the addition of *β*-CD. This might be due to the nonuniform distribution of excess *β*-CD, which finally existed in the form of a small number of crystals [24]. This further indicated that the mixing of PA and *β*-CD was only physical, and the cavity structure of *β*-CD was retained on the nanofiber, which was beneficial to the adsorption of this composite material.

### 3.3. Effect of Initial Concentration of Cationic Dye Solution on the Adsorption of Nanofibers

**Figure 6:**
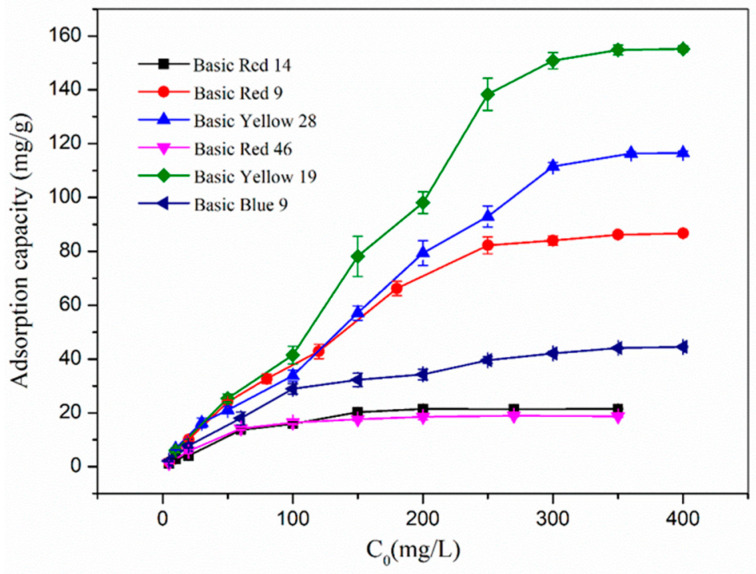
The maximum adsorption capacity of various dyes under different initial concentrations.

The initial concentration of cationic dye solution was an important factor in influencing the adsorption rate and the maximum equilibrium adsorption amount. The relationship between the adsorption amount of six cationic dyes and their initial concentrations are shown in Figure 6. It can be seen from the diagram that the unit adsorption capacity increases with the initial concentration until reaching equilibrium. Therefore, the maximum adsorption capacity of the optimized electro-spun fibers for basic red 9, basic red 14, basic red 46, basic blue 9, basic yellow 19 and basic yellow 28 is 86.71 mg/g, 21.513 mg/g, 18.926 mg/g, 44.525 mg/g, 116.516 mg/g and 155.206 mg/g, respectively.

From the molecular structure of dyes, the effect of electrostatic attraction due to the charge carried by the molecules being adsorbed by the *β*-CD/PA nanofiber membrane was similar. A more significant influence should stem from the molecular size and the length of the flexible side chains, which resulted in steric hindrance. The top three dyes in terms of adsorption capacity, i.e., basic yellow 19, basic yellow 28, and basic red 9, all possessed relatively compact molecular structures, moderate sizes, and relatively short flexible side chains with low steric hindrance. Basic blue 9 with a moderately rigid main chain structure was slightly affected by the side chains, thereby demonstrating a medium adsorption capacity. However, basic red 14 and basic red 46, which had stronger molecular flexibility and more side chains, brought about the lowest adsorption capacity. All these factors suggest that, in the process of adsorbing cationic dyes by the *β*-CD/PA nanofiber membrane, the inclusion of dye molecules by *β*-CD should play a principal role.

### 3.4. Effect of pH on the Adsorption of Nanofibers and the Adsorption Mechanism

**Figure 7:**
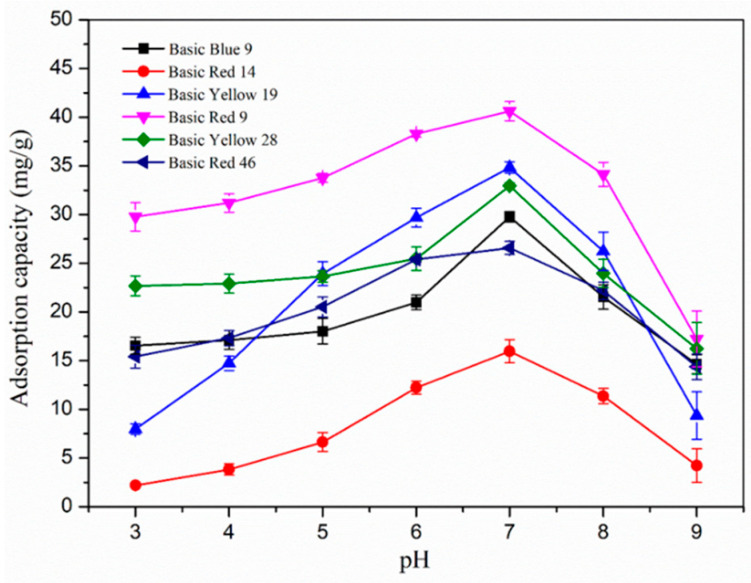
Effect of initial pH value on the adsorption capacity of *β*-CD/PA nanofiber membrane for six cationic dyes.

The pH value was another important reason to affect the adsorption process because the concentration of hydrogen ion (H^+^) would influence the surface charge of an adsorbent and the ionization behaviors of both the adsorbent and dyes [27]. In the range of pH from 3 to 9, the effect of pH value on the adsorption process is shown in Figure 7. It can be seen that the adsorption amount of the nanofiber membrane for cationic dyes increased with the increase of pH, and the most attractive adsorption appeared when the dye solution was neutral. Lower pH values provided more positive charges, which would generate stronger repulsive interaction with the positive groups in a dye molecule and the hydroxyl groups with positive charge on cyclodextrin. Protonated dye molecules were not conducive to forming the host–guest inclusion complexes with protonated *β*-cyclodextrin [28,29]. In addition, because of the high concentration of H^+^, the competitive adsorption with carboxyl groups contained in polyacrylate between the available H^+^ and dye molecules would also decrease the adsorption capacity. As the pH increased, the positively charged groups in the dye molecules gradually became neutral, which enhanced the interaction among the dye molecules with *β*-cyclodextrin, and more activated deprotonation carboxyl groups could form an electrostatic interaction with the dye molecules [2]. When the solution was neutral or alkaline, the carboxylic acid of polyacrylate would interact with the hydroxyl ion (OH^−^), and the adsorption performance would decrease because of the electrostatic interaction between the dye molecules and the absorbent material. As the alkalinity of the dye solution increased further, part of the *β*-cyclodextrin dissolved in the aqueous solution and the adsorption material was damaged, resulting in a significant decrease in adsorption performance.

**Figure 8:**
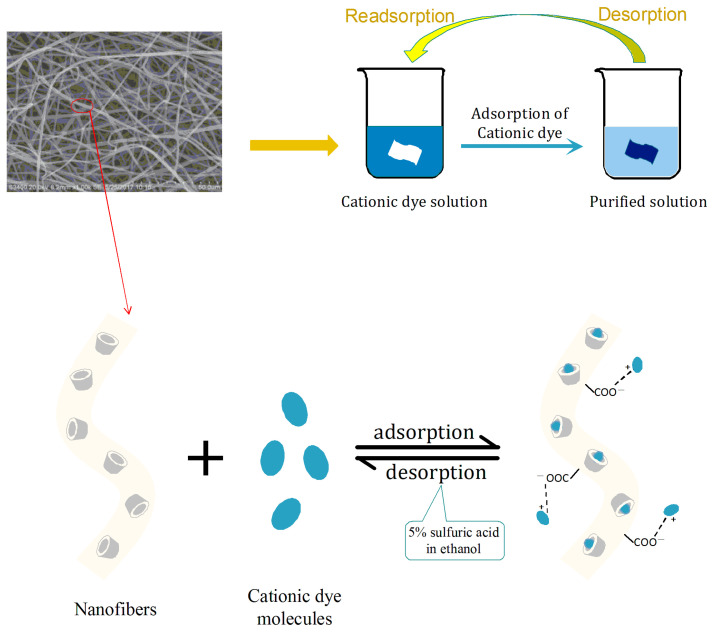
The structure of *β*-CD/PA composite fiber and its adsorption mechanism for cationic dyes.

As the pH value decreased, the adsorption capacity of the fiber gradually decreased, which provided an opportunity for the desorption of the adsorbent. The possible dynamic adsorption process of the nanofiber is shown in Figure 8. In short, the adsorption performance of nanofiber membrane for dye molecules can be attributed to two processes: electrostatic interactions and the host–guest inclusion complexes. For electrostatic interactions, positively charged dye groups could be attached to *β*-CD/PA nanofibers through positive and negative electrostatic interactions with carboxyl groups in polyacrylate. For inclusion cooperation, the cavity of *β*-cyclodextrin in *β*-CD/PA fibers could form a host–object inclusion compound with dye molecules. Based on the above adsorption mechanism, *β*-CD/PA fibers possessed favorable adsorption capacity for cationic dyes.

### 3.5. Adsorption Kinetics of β-CD/PA Nanofiber for Cationic Dyes

**Figure 9:**
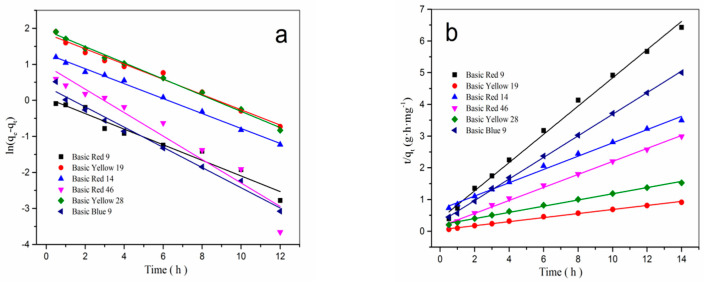
Adsorption kinetic curves of *β*-CD/PA nanofiber for six cationic dyes. (**a**) pseudo-second-order kinetics; (**b**) pseudo-first-order kinetics.

In order to understand the dynamics of the adsorption process, the adsorption kinetics of *β*-CD/PA fibers for six cationic dyes were studied using two kinetic models, the pseudo-first-order model and the pseudo-second-order model. The common equation for the pseudo-first-order kinetic model is [18]:

(2) $$Ln\left(q_{e}-q_{t}\right)=Ln\left(q_{e}\right)-k_{1}t$$

where *q*_e_ and *q*_t_ are the amounts of dye adsorbed (mg/g) at equilibrium and at any instant of time *t* (h), respectively, and *k*_1_ is the rate constant of pseudo-first-order adsorption (h^−1^). The plot of *Ln*(*q*_e_ − *q*_t_) versus *t* gave a straight line for the pseudo-first-order adsorption kinetics, as shown in Figure 9a, and the values of the pseudo-first-order rate constants *k*_1_ and *q*_e_ are given in Table 2.

The equation for the pseudo-second-order kinetic model is as follows [18]:

(3) $$t/q_{t}=1/\left({k_{2}q_{e}}^{2}\right)-1/q_{e}$$

where *q*_e_ is the equilibrium adsorption capacity (mg/g), *q*_t_ is the amount of dye adsorbed at time *t* (mg/g), and *k*_2_ is the rate constant of pseudo-second-order adsorption (g·mg^−1^·h^−1^). Figure 9b is a pseudo-second-order kinetic model line of the adsorption rate for six cationic dyes, and its kinetic parameters are shown in Table 2. Comparing the pseudo-first-order and pseudo-second-order kinetic parameters, the correlation coefficient (*R*^2^) of the pseudo-second-order model is larger, which indicates that the adsorption process of the cationic dyes in the *β*-CD/PA fibers was closer to the pseudo-second-order kinetic model.

### 3.6. Desorption and Recycling Ability

**Figure 10:**
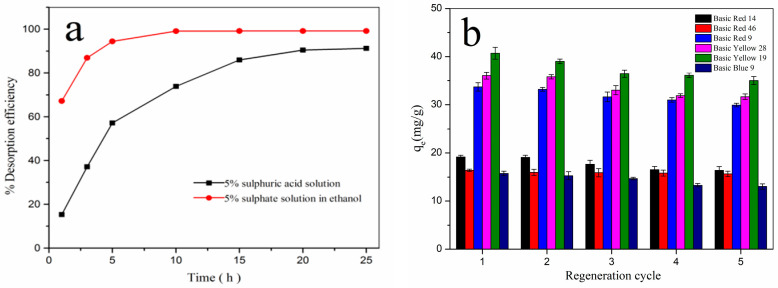
(**a**) Desorption of different desorption fluids for basic yellow 28; (**b**) cyclic adsorption capacity of *β*-CD/PA nanofiber membrane adsorbent.

From the perspective of economic efficiency, the desorption and recycling of adsorbents is vital for the adsorption process. Therefore, two kinds of desorption liquids, 5% sulfuric acid aqueous solution and 5% sulphate alcohol solution (ethanol volume accounted for 60% of the total volume) were used to understand the desorption capacity of the adsorbent materials. The latter obtained better desorption performance, which reached desorption equilibrium after desorption for 10 h, and the desorption rate was 99.13% (Figure 10a). This may be because the solubility of *β*-CD and cationic dyes in ethanol was smaller than their solubility in water, so the cationic dyes adsorbed on the *β*-CD/PA nanofiber membrane by coating were more difficult to get rid of the cavity binding of *β*-CD during the analysis process but would be resolved together with *β*-CD under the action of sulfuric acid. The desorption in this case would be higher.

Using 5% sulphate alcohol solution as the desorption solution, the adsorption and desorption cycles of six cationic dyes were studied, and the adsorption amount of the adsorbent is shown in Figure 10b. The results show that the adsorption capacity for six cationic dyes after five cycles was higher than 85% of the original adsorption effect. These showed that the *β*-CD/PA nanofiber adsorbent had good recycling performance

## 4. Conclusions

Our research has demonstrated a convenient and feasible way to prepare a *β*-CD/PA composite nanofiber membrane adsorbent by electrostatic spinning. Cationic dyes pollutants in water were obviously removed by the synergistic adsorption of *β*-cyclodextrin and polyacrylate in this nanofiber membrane. The adsorption properties of the adsorbent materials under different initial concentrations and pH conditions were studied, and it was found that the adsorbent performed better under high initial concentration and a neutral condition. The adsorption kinetics for cationic dyes showed that the adsorption process could be consistent with the pseudo-second-order kinetic model. In addition, nanofiber membrane adsorbent had good desorption after adsorbing cationic dyes and a high recycling efficiency. These results demonstrated that *β*-CD/PA nanofiber membrane could be used as a promising adsorbent for dye wastewater treatment.

## Figures and Tables

**Table 1:** Details of 5 kinds of electrospinning solutions and fibers.

Sample	PA/g	*β*-CD/g	DMF/mL	(*m_β_*_-CD_/*m*_PA_)/%	Conductivity/μS·cm^−1^	Viscosity/mPa·s	Fiber’s Diameter/μm
a	2	0	16	0	138.9	143.3	25
b	2	0.5	16	25	125.1	154.1	12.2
c	2	1	16	50	106.3	168.5	11.3
d	2	1.5	16	75	94.3	187.5	10.8
e	2	2	16	100	72.7	235.0	11.8

**Table 2:** Kinetics parameters for the adsorption of *β*-CD/PA fibers for six cationic dyes.

Cationic Dyes	Pseudo-First-Order Kinetic			Pseudo-Second-Order Kinetic		
	*q*_e_	*k*_1_	*R* ^2^	*q*_e_	*k*_2_	*R* ^2^
Basic Red 9	2.11	0.2165	0.958	2.18	0.4452	0.995
Basic Yellow 19	14.78	0.2116	0.983	15.26	0.0639	0.997
Basic Red 14	3.71	0.2073	0.995	4.01	0.2095	0.996
Basic Red 46	4.66	0.3256	0.925	4.68	0.2050	0.995
Basic Yellow 28	8.73	0.2245	0.995	9.16	0.0978	0.997
Basic Blue 9	2.75	0.2807	0.983	2.80	0.3402	0.999

## Data Availability

Data are contained within the article.
